# Cleanliness in the Root Canal System: An Scanning Electron Microscopic Evaluation of Manual and Automated Instrumentation using 4% Sodium Hypochlorite and EDTA (Glyde File Prep)―An *in vitro* Study

**DOI:** 10.5005/jp-journals-10005-1073i

**Published:** 2010-09-15

**Authors:** Deepak Raisingani, GK Meshram

**Affiliations:** 1 Associate Professor, Department of Conservative Dentistry and Endodontics, Mahatma Gandhi Dental College and Hospital, Jaipur Rajasthan, India; 2 Professor, Department of Conservative Dentistry and Endodontics, Government Dental College and Hospital, Nagpur Maharashtra, India

**Keywords:** SEM, Automated instrumentation, EDDA.

## Abstract

**Aim :** To assess the cleaning efficacy of manual and automated instrumentation using 4% sodium hypochlorite singly and in combination with Glyde file Prep as root canal irrigant.

**Methodology :** The study utilized 40 extracted human permanent premolars with single, straight and fully formed root. The teeth were then divided into four groups of ten each, Group I and II were prepared by manual instruments with 4% sodium hypochlorite used as irrigant singly [Group I] or in combination with Glyde file prep. Group III and IV were prepared by automated instruments at 250 rpm with 4% sodium hypochlorite as irrigant singly [Group III] and in combination with glyde file prep [Group IV] automated instrumentation. After completion of the root canal preparation the canal, teeth were prepared for SEM examination. These photomicrographs were qualitatively evaluated using criteria. Overall cleanliness, presence or absence of the smear layer, presence or absence of the debris, patency of the opening of dentinal tubules.

**Results :** When comparing the cleansing efficacy of manual and automated instrumentation using 4% sodium hypochlorite better cleansing was there with manual instrumentation. When comparing the cleansing efficacy of manual and automated instrumentation using combination regime cleansing is better with automated instrumentation. When comparing the cleansing efficacy of manual instrumentation using 4% sodium hypochlorite singly and in combination with EDTA, the combination regime led to better cleansing. When comparing the cleansing efficacy of automated instrumentation using 4% sodium hypochlorite singly and in combination regime lead to better cleansing.

**Conclusion :** Neither of instrumentation technique, nor irrigating regimes were capable of providing a completely clean canal. Automated instrumentation with a combination of sodium hypochlorite & EDTA resulted the best cleansing efficacy.

## INTRODUCTION

Endodontic therapy involves a series of procedures that begins with adequate knowledge of biology of pulp and periapical tissue and ends with the subsequent evaluation of the treatment. The equally important intermediate steps consist of biomechanical preparation, which includes direct access for cleaning, shaping and disinfecting root canal.^1^ Root canal therapy was until the turn of the century concerned mainly with the removal pulp tissue and alleviating pain. If the tooth was comfortable, the treatment was considered a success. At that time cleaning and shaping were difficult procedures because instruments available were poorly suited for the task.

The most time consuming and difficult aspect of root canal therapy involves the mechanical cleaning and shaping. Until 1960, root canal instruments were produced of carbon-steel, nowadays stainless steel alloys are the most commonly used. It is said that stainless steel alloys as compared with older carbonsteel alloys can be sterilized largely without detrimental physical changes (considerable corrosion damage). Wein et al reported that the enlargement of root canal by stainless steel files and reamers results in unwanted alteration in the canal shape such as apical transportation (zipping) ledge formation and sometimes even perforation. These procedural errors have a common genesis, which occurs due to stiffness of stainless steel alloys with an inherent tendency to straighten.^2^

In the past two decades, the attention has focused on a family of alloys known as the “shape memory alloys”. “These have remarkable physical properties considered one of the unique properties of pseudoelasticity or superelasticity that has been described as a phenomenon by which material recovers from the induced “plastic strain” on unloading the force and then returning to its original shape. Nickel titanium alloy “Ni-Ti” with a very low modulus of elasticity (~30 Gpa versus ~200 Gpa for stainless steel alloy), superior flexibility in bending and greater resistance to torsional fracture was the ultimate answer to problems associated with stainless steel instruments.^3^ Since most hand preparation techniques are time consuming, technically demanding and show unpredictable outcome, attention has been directed towards automated methods of canal preparation. With the advent of Nickel titanium files the idea of a safe rotary file was born, attempts to use conventional stainless steel files for mechanical instrumentation of root canal have been ongoing for many years with little success. The advantage of this type of instrumentation includes increased debris removal, better shaping, faster and smoother canal preparation, decreased operator fatigue.^4^

Unfortunately, root canals are irregular, complicated systems. Instrumentation alone is inadequate because pulp tissue remnants, debris and root shaving are left behind in the canal. Irrigants play a major role in biomechanical preparation of root canals. Irrigation solution should facilitate the removal of pulp tissue remnants, loose debris and lubricate the canal^5^. Root canals were not irrigated until the middle of1940, after Grossman and Meimann demonstrated the solvent action of sodium hypochlorite on pulp tissue, until then the enlargement of root canal was accompanied by files and reamers in a dry canal. Following the use of sodium hypochlorite for dissolving pulp remnants, it seemed logical to wash out debris, Grossman et al recommended irrigation of sodium hypochlorite and hydrogen peroxide using two solutions alternatively to create an effervesce that helped float debris.^6^ Sodium hypochlorite has been accepted as an irrigant in endodontic over the years, however organic tissue solvent such as sodium hypochlorite used alone has shown to be ineffective in the removal of smear layer. Due to its consistency, viz. it contains both organic and inorganic components, smear layer removed by sodium hypochlorite (organic tissue solvent) is inefficient; for this reason various investigators have investigated chelating agents for removal of smear layer during root canal preparation. Nygaard– Ostby first reported the use of Ethylene diamine tetra acetic acid (EDTA) in endodontic therapy. EDTA is often suggested as an irrigating solution because it has capability to chelate and remove the mineralized portion of smear layer,^1^ however EDTA has shown to be not effective in removing soft tissue. To date, no single irrigant has been demonstrated to be capable of dissolving the organic pulpal material and predentine as well as demineralizing the inorganic calcified portion of canal wall. There have been many studies for and against the manual and automated instrumentation of root canal. The aim of this scanning electron microscopic study is to evaluate cleanliness in the root canal system following hand instrumentation, using stainless steel files compared with engine driven Nickel titanium files using sodium hypochlorite singly and in combination with EDTA as a root canal irrigant.

## MATERIALS AND METHODS

A sample of 40 extracted human permanent premolars with single, straight and fully formed root were selected for the study. These teeth were collected from Department of Oral and Maxillofacial Surgery, Government Dental College and Hospital, Nagpur. They were scrubbed, washed under running water and stored in normal saline at room temperature.

To facilitate the sectioning of teeth for scanning electron microscopic examination (SEM), all the teeth were grooved around the cervical margin of the crown and on the buccal and lingual side of the root by carborundum disk. Adequate care was taken to prevent damage to the root canal surface of the teeth. Conventional endodontic access preparation was done using air turbine hand piece with a round bur BR-46*, extension of the access cavity was achieved with a tapered fissure bur TF-21*. Before instrumentation, the full working length of root canal was established by substracting 1 mm from the actual canal length, which was determined by inserting a size 10-K file until the tip of file was just visible at apical foramen. The teeth were then divided into four groups of ten each, Group I and II were prepared by manual instruments [K-files, Dentsply, Maillefer, Switzerland] with 4% sodium hypochlorite used as irrigant singly [Group I] or in combination with Glyde file prep (Ethylene diaminetetraacetic acid (EDTA) and carbamide. {Group II} Manual instrumentation was done using a crown down technique.

Group III and IV were prepared by automated instruments [Endodontic contra-angle torque control hand piece, Anthogyr, France, 1:64 with nickel titanium file] at 250 to 400 rpm with 4% sodium hypochlorite as irrigant singly [Group III] and in combination with glyde file prep [Group IV] automated instrumentation was done using a crown down technique.

After completion of the root canal preparation, the canal was irrigated with sterile water to terminate any solvent action of the irrigant and to remove any precipitate that may have been formed from the irrigant. All canals were dried with paper points.

**Fig.1:**
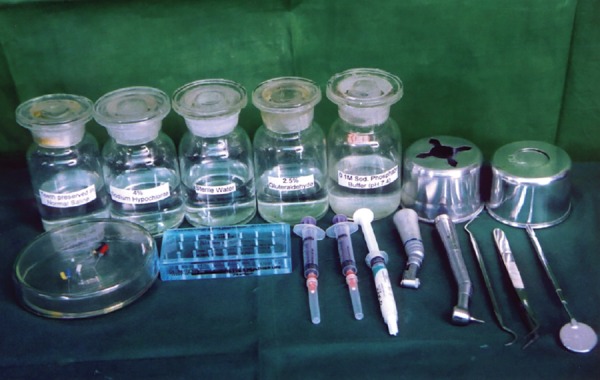
Armamentarium From left to right: First row teeth preserved in saline, 4% sodium phosphate, buffer, waste receiver, cotton holder Second row: Manual instruments, air-rotor burs, irrigating syringes, gylde file prep, micromotor hand piece, air turbine hand piece, dental explorer, twiser, mouth mirror

**Fig.2:**
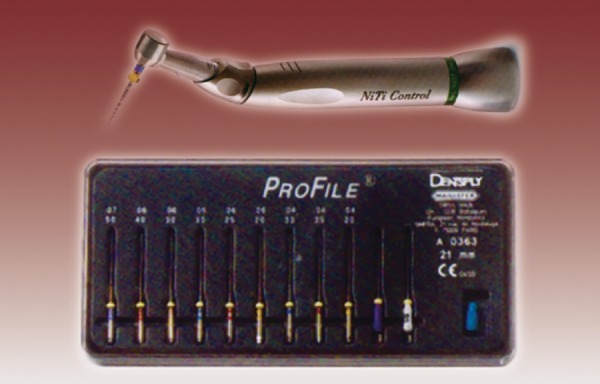
Endodontic contra-angle torque control handpiece anthogyr, with nickel titanium file (Profiles)

**Fig. 3:**
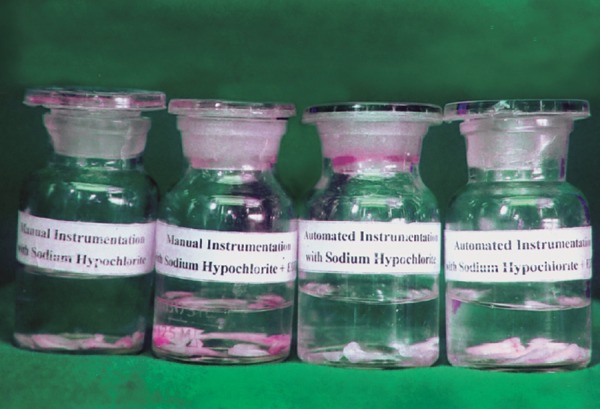
Specimens’ stored in 2.5% gluteraldehyde prepared in 0.1 M sodium phosphate buffer

## SCANNING ELECTRON MICROSCOPIC EXAMINATION

Complete surface of the specimen was scanned. Photomicrographs were taken at apical, middle and coronal one-third at a magnification 1000X and 2000X. These photomicrographs were qualitatively evaluated using following criteria.

1. Overall cleanliness
2. Presence or absence of the smear layer
3. Presence or absence of the debris
4. Patency of the opening of dentinal tubules.

These photomicrographs were graded on a scale of I-IV as described below.

Grade I           Clean without debris and smear layer presence of patent dentinal tubules.

Grade II           Mild debris and smear layer, many patent dentinal tubules.

Grade III           Moderate debris and smear layer, few patent dentinal tubules.

Grade IV          Severe debris and smear layer, no patent dentinal tubules.

The data on the score level was recorded directly onto coding sheet. The statistical analysis was carried out by means of Mann-Whitney rank sum test was conducted using Sat version 7.0, P was set to < 0.05. The aim was to assess any statistically significant difference between the cleanliness efficacy of manual and automated instrumentation using sodium hypochlorite singly and in combination with EDTA.

**Fig. 4:**
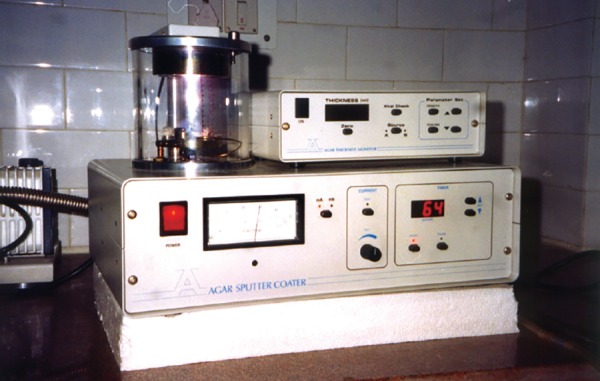
Sputter coating unit (Agar auto coating unit, Essex, UK)

**Fig. 5:**
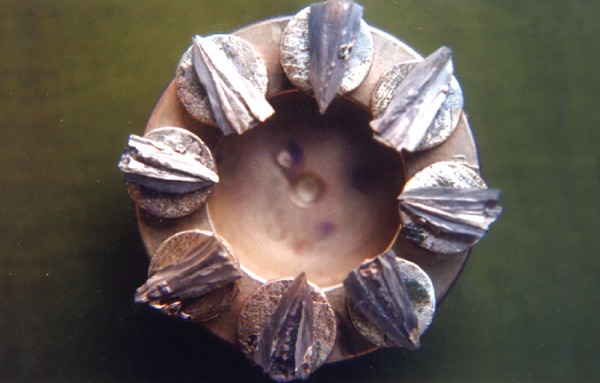
Sputter coated specimens mounted on metal stubs

**Fig. 6:**
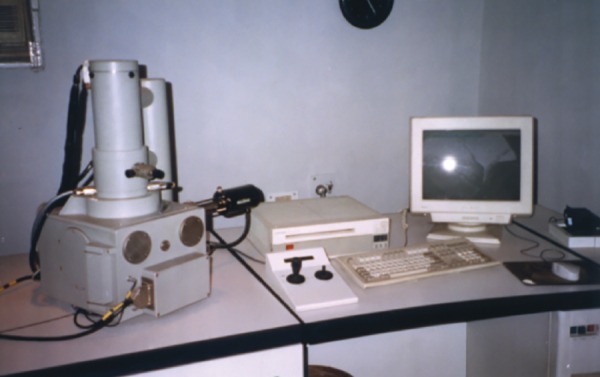
Scanning electron microscope (LEO-electron microscope Cambridge, England)

## OBSERVATION AND RESULTS

Table 1 shows that 10 specimens were studied for cleansing efficacy at three different levels. Thus, 30 photomicrographs were obtained for this study.

None of the photomicrographs showed grade I cleansing efficacy.

**Table 1:** **Table 1:** Manual instrumentation with sodium hypochlorite as an irrigant (Group I)

*Teeth no.*		*MI apical*		*MI mid*		*MI coronal*	
1		III		IV		II	
2		II		III		II	
3		II		III		III	
4		III		II		III	
5		III		II		II	
6		III		III		II	
7		II		IV		III	
8		II		II		III	
9		II		III		III	
10		III		III		III	

At the cervical one-third level out of 10 photomicrographs six showed Grade III cleansing, four showed Grade II cleansing.

At middle one-third, two photomicrographs showed Grade IV cleansing, five showed Grade III cleansing, three showed Grade II cleansing.

At apical one-third, five photomicrographs showed Grade III cleansing, five showed Grade II cleansing.

Thus, out of 30 photomicrographs, seven showed Grade II cleansing, 16 showed Grade III cleansing, Seven showed Grade IV cleansing.

Table 2 shows that 10 specimens were studied for cleansing efficacy at three different levels, thus 30 photomicrographs were obtained for this study.

**Table 2:** **Table 2:** Manual instrumentation with 4% sodium hypochlorite in combination with EDTA as an irrigant (Group II)

*Teeth no.*		*MI apical*		*MI mid*		*MI coronal*	
1		III		II		I	
2		II		I		I	
3		II		I		II	
4		I		II		II	
5		II		III		II	
6		II		III		II	
7		III		II		I	
8		I		II		II	
9		II		I		I	
10		II		II		II	

At the cervical one-third out of 10 photomicrographs, four showed Grade I cleansing, six showed Grade II cleansing.

At the middle one-third, three photomicrographs showed Grade I cleansing, five showed Grade II cleansing, two showed Grade III cleansing.

At the apical one-third, two photomicrographs showed Grade I cleansing, six showed Grade II cleansing, two showed Grade III cleansing.

Thus, out of 30 photomicrographs, nine showed Grade I cleansing, 17 showed Grade II cleansing, four showed Grade III cleansing, none of photomicrograph showed Grade IV cleansing.

Table 3 shows that 10 specimens were studied for cleansing efficacy at three different levels. Thus, 30 photomicrographs were obtained for this study.

At coronal level, out of 10 photomicrographs, one showed Grade II cleansing, five showed Grade III cleansing, four showed Grade IV cleansing.

At middle one-third level, one photomicrograph showed Grade II cleansing, four showed Grade III cleansing, five showed Grade IV cleansing.

At apical one-third, five photomicrographs showed Grade III cleansing, five showed Grade IV cleansing.

Thus, out of 30 photomicrographs none showed Grade I cleansing, two showed Grade II, 14 showed Grade III cleansing, 14 showed Grade IV cleansing.

Table 4 shows that 10 specimens were studied for cleansing efficacy at three different levels. Thus, 30 photomicrographs were obtained for this study.

At the cervical level, out of 10 photomicrographs, seven showed Grade I cleansing, two showed Grade II cleansing, one showed Grade III cleansing.

At the middle one-third level, out of 10 photomicrographs, six photomicrographs showed Grade I cleansing, four showed Grade II cleansing.

**Table 3:** **Table 3:** Automated instrumentation with 4% sodium hypochlorite as an irrigant (Group III)

*Teeth no.*		*AI apical*		*AI mid*		*AI coronal*	
1		III		IV		II	
2		III		III		IV	
3		IV		III		III	
4		IV		IV		III	
5		III		IV		IV	
6		III		III		IV	
7		III		III		III	
8		IV		II		III	
9		IV		IV		IV	
10		IV		IV		III	

At the apical one-third level, out of 10 photomicrographs five showed Grade I cleansing, four showed Grade II cleansing, one showed Grade III cleansing.

Thus, out of 30 photomicrographs, 18 showed Grade I cleansing, 10 showed Grade II cleansing, two showed Grade III cleansing, none showed Grade IV cleansing.

The data shown in Tables 1 to 4 are analyzed Mann-Whitney Rank Sum Test was conducted using Sat version 7.0, P was set to < 0.05.

It was evident that when comparing the cleansing efficacy of manual and automated instrumentation using 4% sodium hypochlorite, better cleansing was there with manual instrumentation and there is statistically significant difference overall P_total_ 0.0069, apical P_apical_ 0.0021 and coronal level P_coronal_ 0.0063 and a nonsignificant difference at middle one third P_mid_ 0.1316.

When comparing the cleansing efficacy of manual and automated instrumentation using 4% sodium hypochlorite with EDTA, cleansing is better with automated instrumentation, however there is statistically nonsignificant difference overall P_total_ 0.0732, at middle one-third P_middle _0.0537 and coronal one-third P_coronal_ 0.2514. There exist a statistically difference at apical one-third level P_apical_ 0.0202.

When comparing the cleansing efficacy of manual instrumentation using 4% sodium hypochlorite singly and in combination with EDTA, the combination regime led to better cleansing and there is statistically significant difference overall P_total_ 0.0014, at middle one-third P_mid _0.0116, coronal one-third level P_coronal_ 0.0014. There exist a statistically nonsignificant difference of apical one-third P_apical_ 0.0898.

When comparing the cleansing efficacy of automated instrumentation using 4% sodium hypochlorite singly and in combination with EDTA automated instrumentation with 4% sodium hypochlorite + EDTA lead to better cleansing and there is statistically highly significant difference. Overall P_total_ 0.0002, and apical P_apical_ 0.0001, middle one-third P_middle_ 0.0001, and cervical level P_cervical_ 0.0003.

**Table 4:** **Table 4:** Automated instrumentation with 4% sodium hy-pochlorite in combination with EDTA as root canal irrigant (Group IV)

*Teeth no.*		*AI apical*		*AI mid*		*AI coronal*	
1		I		II		I	
2		I		II		I	
3		II		I		I	
4		III		I		II	
5		I		I		I	
6		II		II		I	
7		II		I		I	
8		I		I		III	
9		I		I		II	
10		II		II		I	

**Fig.7:**
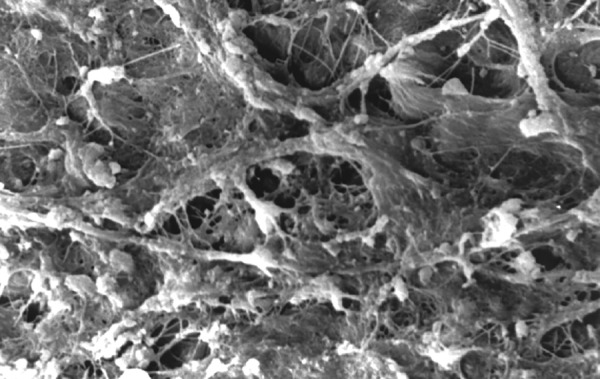
Photomicrograph (x2000 magnification) of uninstrumented specimen middle one-third

**Fig.8:**
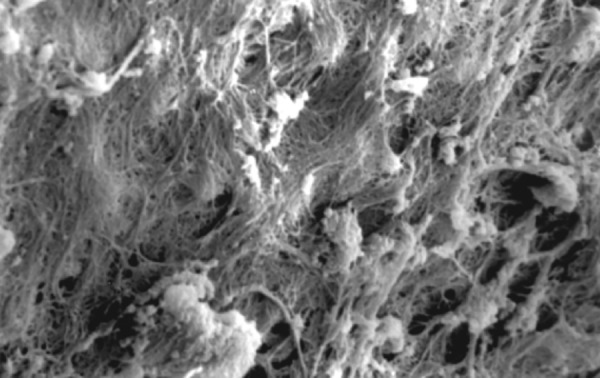
Photomicrograph (x2000 magnification) of uninstrumented specimen-cervical one-third

**Fig.9:**
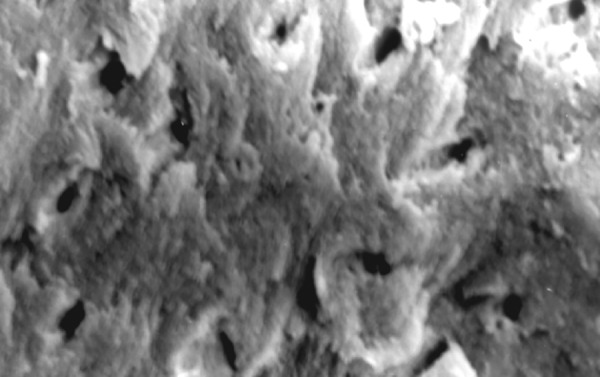
Photomicrograph (x2000 magnification) of manual instrumented specimen with sodium hypochlorite-apical one-third

**Fig.10:**
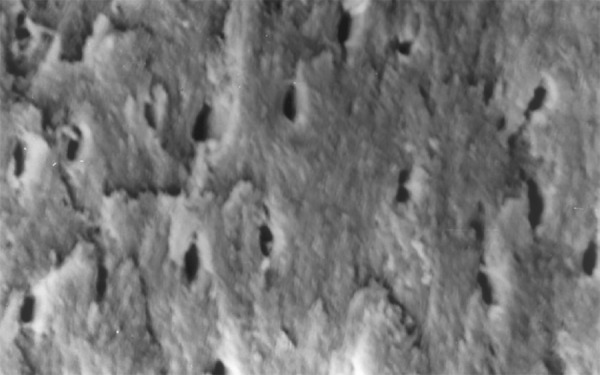
Photomicrograph (x2000 magnification) of manual instrumented specimen with sodium hypochlorite middle one-third

**Fig.11:**
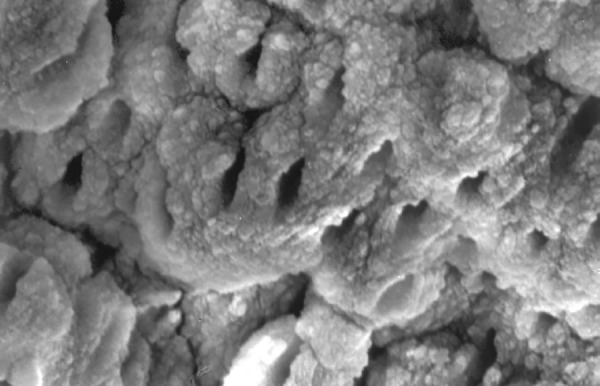
Photomicrograph (x2000 magnification) of manual instrumented specimen with sodium hypochlorite-cervical one-third

**Fig.12:**
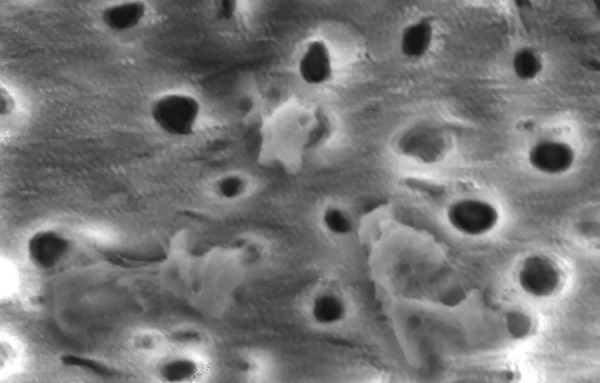
Photomicrograph (x2000 magnification) of manual instrumented specimen with sodium hypochlorite and EDTA-apical one-third

**Fig.13:**
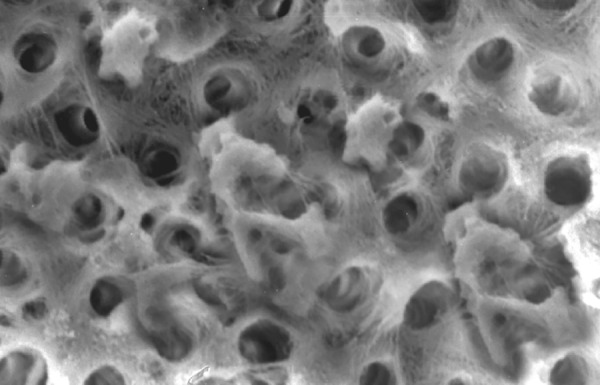
Photomicrograph (x2000 magnification) of manual instrumented specimen with sodium hypochlorite and EDTA-middle one-third

**Fig.14:**
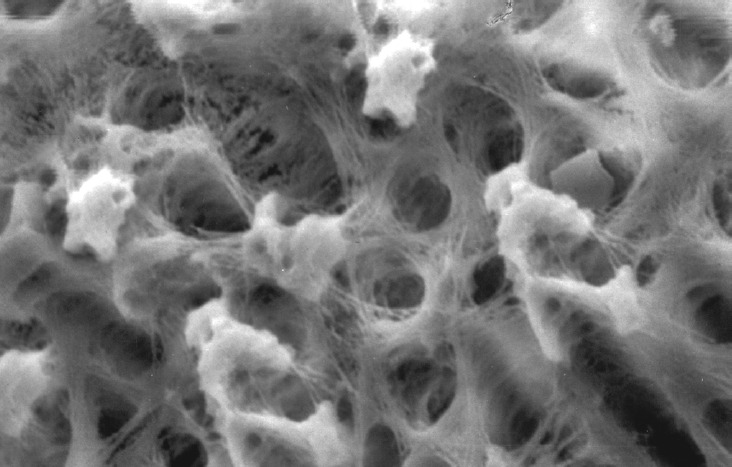
Photomicrograph (x2000 magnification) of manual instrumented specimen with sodium hypochlorite and EDTA-Cervical one-third

**Fig.15:**
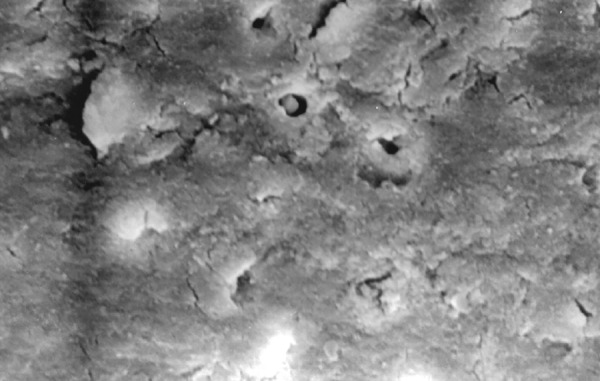
Photomicrograph (x2000 magnification) of automated instrumented specimen with sodium hypochlorite-apical one-third

**Fig.16:**
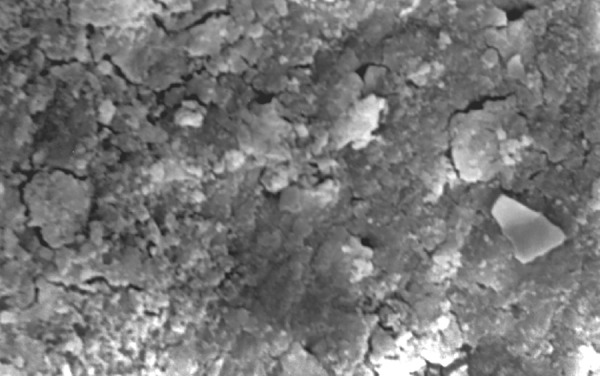
Photomicrograph (x2000 magnification) of automated instrumented specimen with sodium hypochlorite-middle one-third

**Fig.17:**
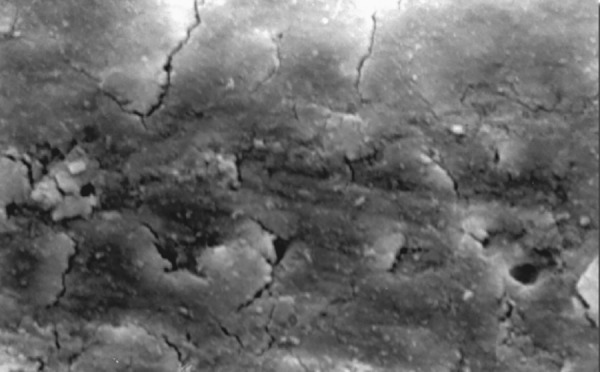
Photomicrograph (x2000 magnification) of automated instrumented specimen with sodium hypochlorite-cervical one-third

**Fig.18:**
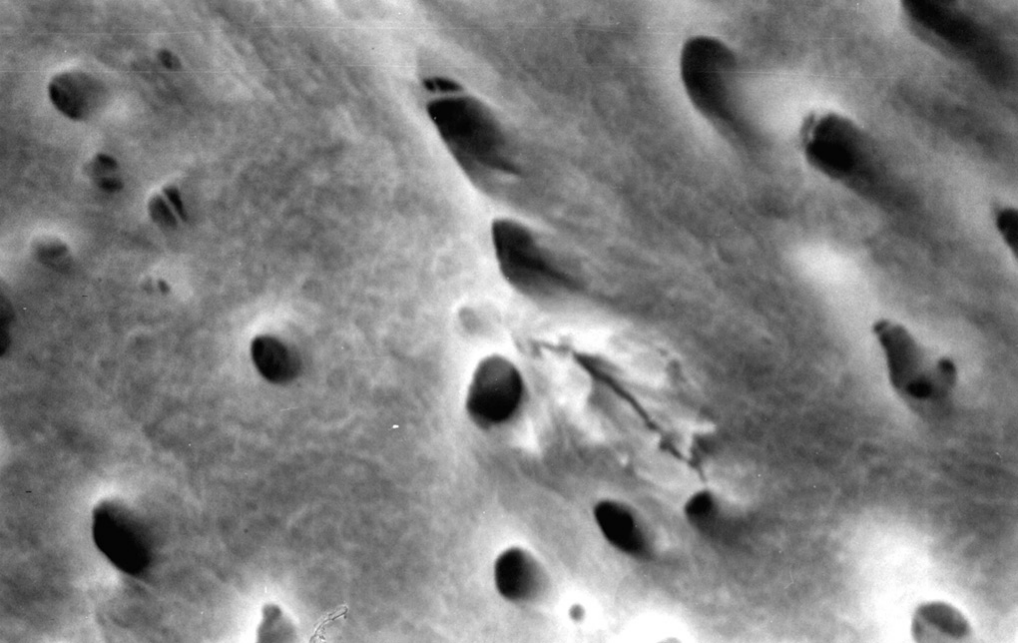
Photomicrograph (x2000 magnification) of automated instrumented specimen with sodium hypochlorite and EDTA-apical one-third

**Fig. 19:**
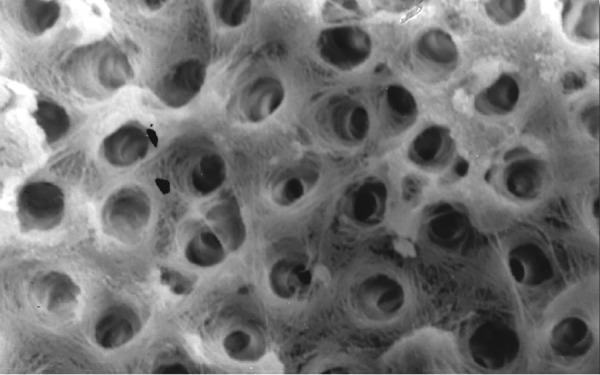
Photomicrograph (x2000 magnification) of automated instrumented specimen with sodium hypochlorite and EDTA-Middle one-third

**Fig. 20:**
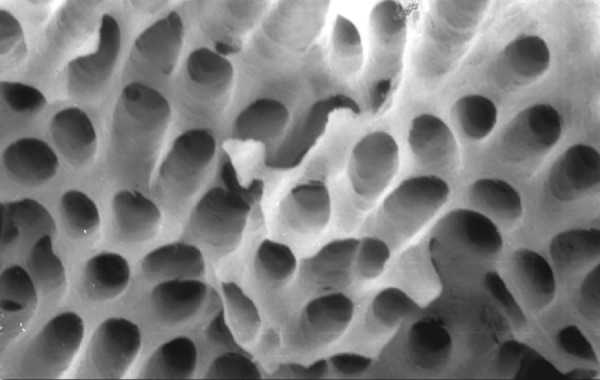
Photomicrograph (x2000 magnification) of automated instrumented specimen with sodium hypochlorite and EDTA-Cervical one-third

## DISCUSSION

Emphasis in endodontic research has been placed for some years now on the mechanical aspect of cleaning and shaping the root canal system. This is in contrast to an earlier long standing preoccupation with chemical methods. There is currently a swing of the endodontic pendulum back to a reconsideration of chemical adjuvant. There is the important difference, however present interest lies in facilitating both the mechanical and chemical methods for root canal cleaning and shaping (chemomechanical preparation).

The demands encountered in cleaning and shaping of the root canal system have fostered the development of many new technologies aimed to facilitate this process. There are enormous difference in opinion regarding the best method for preparing the root canal system. Till recently, root canal preparation was done with stainless steel files using sodium hypochlorite as an irrigant. Stainless steel alloys had replaced the older carbon-steel alloys for the manufacture of endodontic instruments as Stenman et al observed the effect of sterilization on the cutting efficiency and resistance to fracture. Their results demonstrated that sterilization can lead to considerable corrosion damage to carbon steel instruments, whereas no significant difference on the mechanical properties was observed on stainless steel instruments.^4^ However, Wein et al reported that root canal preparation with stainless steel files lead to apical transportation (zipping) and an hour glass shape, as a consequence the root canal morphology is adversely affected, a violation of the basic principle that endodontic preparation should retain the original shape of the canal. This makes obtaining a successful apical seal difficult.^2^ In an attempt to reduce procedural errors Walia et al 1988 introduced the first nickel titanium files. The nickel titanium file with a very low modulus of elasticity, superior flexibility and greater resistance to fracture was expected to reduce the procedural errors associated with stainless steel files. With the advent of nickel titanium files the idea of safe automated instrumentation was introduced to decrease instrumentation time, operator fatigue and to simplify root canal preparation.^7^

It has been shown by several investigators that shaping and cleansing of root canal system will not sufficiently clean the complex root canal system. Moreover, instrumentation will produce a smear layer, which is generated whenever dentine is instrumented. Scanning electron microscopic studies have shown that after instrumentation, debris are smeared on the walls of the root canal in the same way as a smear layer covers dentinal surface after cavity preparation with rotary or hand instrumentation. Even the aperture of dentinal tubules are obliterated by plugs of debris.^8^ The exact proportion of endodontic smear layer has not been determined, but it has been shown that its composition is both organic and inorganic. The inorganic material in the smear layer is made up of tooth structure and some inorganic contaminants. The organic components may consist of heated coagulated proteins, necrotic or viable pulp tissue, odontoblastic process plus saliva, blood cells and microorganisms. This smear layer may be 1 to 2 micron thick.^8^

The presence or absence of smear layer in endodontics is important. Several investigators have suggested that smear layer should not be removed, as it plugs the orifices of the dentinal tubules reducing permeability of dentine. This allows the smear layer to act as a protective barrier, which can prevent further bacterial penetration of the tubules. However, other investigators have suggested that smear layer should be removed, as bacteria and bacterial products found in smear layer can provide a reservoir of potential irritants. It also prevents medicaments and filling materials from directly contacting the dentine. Another source of indecisiveness about keeping or removing the smear layer is its long-term stability. The smear layer and its provisional tenacity is a separate structure from the underlying dentine. A situation such as this would be deadly to the foundation of gutta percha obturated over the smear layer. Thus, it is axiomatic that the removal of smear layer permits a better adaptation of sealer and obturating material in dentine.^9^

Sodium hypochlorite is the most commonly used irrigant in root canal treatment and has proven to be an excellent irrigating solution due to its tissue dissolving capability and microcidial activity. According to Moorer and Wesselink (1982) the active principle of sodium hypochlorite solution is the amount of undissociated hypochlorite molecule (NAOCL), which is consumed in the interaction with organic matter. However, its action does not affect inorganic material.^10^ Therefore, the additional irrigation with chelating agents is recommended to remove debris and smear layer. A chelator works by interacting with metallic ions. In case of dentine, it reacts with calcium ions from dentine to produce a metallic chelate, the removal of calcium ions from dentine demineralizes the dentine and aid in the removal of the inorganic components of the smear layer.^11^

Results of the study also show that when comparing manual and automated instrumentation (Group I and Group III) using sodium hypochlorite as an root canal irrigant, smear layer was observed at all three levels, and the amount of smear layer produced by automated preparation was greater (Figs 15 to 17) than that produced by conventional manual instrumentation. (Figs 9 to 11), When comparing the cleansing efficacy of sodium hypochlorite singly with that of combination regime (NaOCl and EDTA) viz. Group I and II, Group III and IV, it has been found out that when a combination of sodium hypochlorite and EDTA has been used, it lead to improved cleansing efficacy and removal of smear layer and opening of the dentinal tubules (Figs 12 to 14 and Figs 18 to 20), as compared to when sodium hypochlorite is used singly (Figs 9 to 11 and Figs 15 to 17). Comparison of Group II and IV shows that regardless of the method of instrumentation, the combination regime of root canal irrigants (EDTA+ sodium hypochlorite) leads to removal of smear layer and open dentinal tubules and left only minimal debris (Figs 12 to 14 and 18 to 20).

The observation of this study is consistent with those Luca DM et al (1996),^5^ Ahlquist M et al (2001)^12^ concluded that rotary root canal instrumentation leads to moderate to heavy smear layer, which no single irrigant was capable of removing. However, a combination of EDTA and sodium hypochlorite effectively removed soft tissue remnants as well as the smear layer.

Baumgartner JC et al (1987),^13^ Blitzkow G et al (1996),^15^ Liolios E et al (1997),^14^ Peters OA et al (2000),^15^ Schafer E et al (2002)^16^ concluded that when sodium hypochlorite was used as the irrigating solution, both manual and automated preparation showed root canal walls with a dense smear layer obscuring the dentinal tubules entrance plus a large amount of debris. The amount of smear layer was more with the automated preparation. However, when root canal walls of teeth were treated with a chelating agent EDTA and a final flush with sodium hypochlorite as an irrigating solution, the root canal appeared extremely clear and smooth.

## SUMMARY AND CONCLUSION

According to the findings and within the limitations of this study, following conclusions were drawn:

Neither instrumentation technique nor irrigating regimes were capable of providing a completely clean canal.

When comparing the cleansing efficacy of manual instrumentation with automated instrumentation using 4% sodium hypochlorite, cleansing was better with manual instrumentation, as automated instrumentation led to root canal walls with a heavy smear layer.

When comparing the cleansing efficacy of manual instrumentation with automated instrumentation using 4% sodium hypochlorite in combination with EDTA, cleansing was better with automated instrumentation, however it was statistically nonsignificant.

When comparing the cleansing efficacy of manual instrumentation using 4% sodium hypochlorite singly to a combination with EDTA, it was found out that the cleansing efficacy was better with combination regime.

When comparing the cleansing efficacy of automated instrumentation using 4% sodium hypochlorite singly to a combination with EDTA, it was found out that the cleansing efficacy was better with the combination regime, as when 4% sodium hypochlorite is used as irrigant, a heavy smear layer was observed on the root canal walls.
